# Fate of *Listeria monocytogenes* on Fresh Apples under Different Storage Temperatures

**DOI:** 10.3389/fmicb.2017.01396

**Published:** 2017-07-24

**Authors:** Lina Sheng, Katheryn Edwards, Hsieh-Chin Tsai, Ines Hanrahan, Mei-Jun Zhu

**Affiliations:** ^1^School of Food Science, Washington State University, Pullman WA, United States; ^2^Washington Tree Fruit Research Commission, Wenatchee WA, United States

**Keywords:** fresh apple, *L. monocytogenes*, survival, storage, fruit decay

## Abstract

Fresh apples are typically stored for up to 1 year commercially; different apple varieties require different storage temperatures to maintain their quality characteristics. There is sparse information available about *Listeria monocytogenes* survival on fresh apples under various storage temperatures. The objective of this study was to comprehensively evaluate the effect of storage temperature on apple fruit decay and *L. monocytogenes* survival. Unwaxed apple fruits of selected varieties (Fuji and Granny Smith) were dip inoculated in a three-strain *L. monocytogenes* cocktail to establish ∼3.5 and 6.0 Log_10_ CFU/apple. Twenty-four hours post-inoculation, apples were subjected to 1, 4, 10, or 22°C storage for up to 3 months. Apples under the different storage treatments were sampled at 1-, 4-, 7- and 14-day for short-term storage under all four tested temperatures, and 2-, 4-, 8-, and 12-week for long-term storage at 1, 4, and 10°C. A set of uninoculated and unwaxed apples were simultaneously subjected to the previously mentioned storage temperatures and sampled biweekly for their total bacterial count (TPC) and yeasts/molds (Y/M) count. During the 2-week short-term storage, *L. monocytogenes* population on organic Granny Smith apples stored at 1, 4, or 10°C was reduced by 0.2–0.3 Log. When apples were stored at 22°C, there was a 0.5–1.2 Log_10_ CFU/apple reduction 14-day post storage dependent on the initial inoculation level. During the 12-week cold storage under 1, 4, and 10°C, *L. monocytogenes* count on organic Granny Smith apples decreased by 0.5–1.5 Log_10_ CFU/apple for both inoculation levels. *L. monocytogenes* had similar survival pattern on conventional Granny Smith and Fuji apples with 0.8–2.0 Log_10_ CFU/apple reduction over a 3-month cold storage period. Interestingly, both TPC and Y/M count were stable regardless of apple variety or cultivation practice during the 12-week storage at all tested temperatures. In summary, while *L. monocytogenes* did not proliferate on apple surfaces during 12 weeks of refrigerated storage, only a limited reduction of *L. monocytogenes* was observed in this study. Therefore, the apple industry cannot rely on cold storage alone to control this pathogen. Additional interventions are needed to eradicate *Listeria* on fresh apples during long-term cold storage.

## Introduction

*Listeria monocytogenes* is a major foodborne pathogen and causes about 1600 illnesses annually in the United States with a mortality rate of ∼16% (Scallan et al., 2011). *L. monocytogenes* is listed by the U.S. Food and Drug Administration as a “pathogen of concern” and has been singled out on ready-to-eat (RTE) produce due to its nature as a environmental species, its frequent occurrence in many produce-associated environment and operations, and its high mortality rate (Scallan et al., 2011). Historically, *L. monocytogenes* outbreaks were associated with RTE meat such as turkey franks (CDC, 1989), hot dog (CDC, 1999), and delicatessen turkey meat (CDC, 2000). Dairy products, such as ice cream (Rietberg et al., 2016) and soft cheese products (Choi et al., 2014; Crowe et al., 2015; Heiman et al., 2016), are frequently involved in *L. monocytogenes* outbreaks. In recent years, increasing numbers of *L. monocytogenes* outbreaks were associated with fresh produce. The 2011 cantaloupe outbreak resulted in 143 hospitalizations and 33 deaths (Cosgrove et al., 2011). The 2014–2015 multistate *L. monocytogenes* outbreak related to caramel apples caused 35 illnesses in 12 states including 7 deaths (CDC, 2015). In addition, there were numerous *L. monocytogenes* recalls related to fresh apples (FDA, 2015a), sliced apples (CFIA, 2015; FDA, 2016), and stone fruits including whole peaches, nectarines, plums, and pluots (Jackson et al., 2015). The above evidence indicates *L. monocytogenes* has become an increasing threat to fresh produce safety and in particular to tree fruit producers.

Unique among other foodborne pathogens, *L. monocytogenes* has the ability to survive and even grow on fresh produce at refrigerated temperatures. *L. monocytogenes* was stable on lettuce during a 5-day storage at 5°C (Poimenidou et al., 2016) and tomatoes during the 20-day storage under 10°C (Beuchat and Brackett, 1991). A recent study indicated that *L. monocytogenes* established on fresh Gala apples were stable during 15-day storage at 5°C; while under the same conditions, the population of *L. monocytogenes* inoculated on caramel apples increased by ∼3 Log_10_ CFU/apple (Salazar et al., 2016). In addition, *L. monocytogenes* population increased by more than 3 Log_10_ CFU/apple in caramel apples with an inserted stick within 3 days of storage at 25°C (Glass et al., 2015; Salazar et al., 2016). These data have corroborated the concerns about *L. monocytogenes* and its negative effect on fresh produce safety. However, inadequate information is available about the behavior of *L. monocytogenes* on fresh apples. The Produce Rule of the Food Safety Modernization Act (FSMA) (FDA, 2015b) requires the fresh apple growers, packers, as well as processors to identify and adopt validated and effective preventive methods to reduce microbial risk in fresh apples by implementing worker training and hygienic practices, control of agricultural water, biological soil amendments, and wild animals, as well as sanitation of equipment, tools, and buildings. This study aimed to evaluate the fate of *L. monocytogenes* in fresh apples stored at different temperatures during both short- and long-term storage. Granny Smith apples (GSA) were studied since it’s a cultivar involved in the *L. monocytogenes* outbreaks (FDA, 2014). Both organic and conventional GSA were used to elucidate the potential influence of different native flora due to the cultivation differences (Camatti-Sartori et al., 2005). Conventional Fuji apples, which have a different surface structure, were also used in this study.

## Materials and Methods

### *L. monocytogenes* Strains and Culture Condition

*Listeria monocytogenes* NRRL B-57618 (1/2a), NRRL B-33053 (4b), and NRRL-33466 (1/2b) were obtained from USDA-ARS culture collection [National Center for Agricultural Utilization Research (NRRL), Peoria, IL, United States] and maintained at -80°C in Trypticase Soy Broth (Becton, Dickinson and Company, Sparks, MD, United States) supplemented with 0.6% Yeast Extract (Fisher Scientific, Fair Lawn, NJ, United States) (TSBYE) and 20% (v/v) glycerol. The frozen culture was first activated in TSBYE at 37°C for 24 h statically, and then 1:500 subcultured in TSBYE for additional 24 h at 37°C before use.

### Inoculum Preparation

Twice-activated bacteria cultures were centrifuged at 8,000 × *g* for 5 min at 4°C. The resulting bacteria pellets were washed once and then re-suspended in Phosphate Buffered Saline (PBS, pH7.4). Equal volume of each *L. monocytogenes* strain was combined to make a three-strain cocktail and, subsequently, diluted to ∼6 and 3 Log_10_ CFU/ml for apple inoculation. At the high inoculation level, there were 6.29 ± 0.04, 6.42 ± 0.04, and 6.01 ± 0.07 Log_10_ CFU/apple on organic GSA, conventional GSA, and Fuji apples, respectively, immediately after inoculation. At the low inoculation level, 3.01 ± 0.06, 3.04 ± 0.07, and 3.48 ± 0.03 Log_10_ CFU/apple on organic GSA, conventional GSA, and Fuji apples, respectively, immediately after inoculation.

### Inoculation of Apples

Whole fresh unwaxed GSA (organic and conventional) and Fuji apples (conventional) were donated by Allan Brothers Inc. and Stemilt Growers LLC. All fruit was of commercial maturity (the optimum maturity for the fresh market or storage) (Girschik et al., 2017). Apples were delivered from the commercial packing houses 1-week prior to inoculation and stored in a walk-in cooler (4°C) temporarily. Medium size apples (with an average weight ∼220 g) without cuts, bruising, or scars were selected and rinsed with cold tap water and dried on a bench top overnight to balance apple temperature to room temperature (RT). The apples were then inoculated with *L. monocytogenes* by submerging 15 apples each time in 5 L of three-strain *L. monocytogenes* cocktail inoculum solution at a selected inoculation level, and gently massaged by turning the apples in the inoculum for 10 min so that microorganisms were evenly distributed in the sample. For each variety at each inoculation level, four inocula were independently prepared with a total of ∼100 apples inoculated per inoculum. Inoculated apples were dried for 24 h at RT (∼22.5°C) before being subjected to storage. Twelve apples were sampled (three apples were randomly sampled from each of four inoculation batches) immediately following inoculation and 24 h post-inoculation for microbial enumeration to confirm inoculum uniformity, inoculation level, and the attachment of *L. monocytogenes* on fresh apples.

### Storage Treatment of Apples

Both uninoculated and 24 h post-inoculation apples were placed in cardboard boxes and stored at 1°C, 4°C, 10°C, and RT in lab-scale refrigerated incubators. Temperatures were monitored continuously by the built-in sensors, and double-checked weekly using two calibrated digital thermometers (Omega HH11, Omega Engineering Inc., Stamford, CT, United States) to ensure precision. Actual storage temperatures were 0.93 ± 0.01, 4.17 ± 0.03, 9.76 ± 0.03, and 22.65 ± 0.03°C. Twelve apples (three apples from each of the four inoculation batches) were sampled for microbial analysis from each temperature of each inoculation level (uninoculated, high and low inoculation levels) at selected sampling points. Uninoculated apples were sampled at 2-, 4-, 8-, 12-week of storage for total plate count (TPC) and yeasts/molds (Y/M) enumeration. Apples inoculated with *L. monocytogenes* were sampled at 1-, 4-, 7- and 14-day intervals for short-term storage, and at 2-, 4-, 8-, and 12-week for long-term cold storage (1, 4, and 10°C).

### Microbial Analysis of Apples

Each apple was placed into a sterile stomacher bag with 10 ml sterile PBS and hand-rubbed for 1.5 min. Wash solutions from each bag were 10-fold serially diluted with sterile PBS, and 0.1 or 1 ml from appropriate dilutions was plated on duplicate appropriate plates. For inoculated apples, TSAYE (TSBYE with 1.5% agar) plates were used for apples with a *L. monocytogenes* level higher than 4.0 Log_10_ CFU/apple where *L. monocytogenes* counts are much higher than the background flora (<3.0 Log_10_ CFU/apple). TSAYE overlaid with Modified Oxford agar (MOX, Becton, Dickinson and Company, Sparks, MD, United States) plates were used for the enumeration of *L. monocytogenes* when its counts are lower than 4.0 Log_10_ CFU/apple to discern from resident background flora of apples. The detection limit of *L. monocytogenes* is 10 CFU/apple. For uninoculated apples, rub solutions were plated onto TSAYE for TPC and Potato Dextrose Agar (PDA, Becton, Dickinson and Company, Sparks, MD, United States) plates for Y/M count. TSAYE plates were incubated at 35 ± 1°C for 48 h. PDA plates were incubated at RT for 5 days.

### Statistical Analysis

Data was analyzed by GLM from Statistical Analysis Systems (SAS, Cary, NC, United States). Mean values were compared by least significant difference (LSD) multiple-comparison test. *P-*values less than 0.05 were considered significant. Four batches of inocula were independently used to inoculate apples at each inoculation level for each variety. Each individual apple was an experimental unit. At each sampling point, three apples were randomly sampled from each batch (*N* = 12). Results were reported as mean ± SEM (standard error of mean).

## Results

### *L. monocytogenes* Survival on Organic GSA

**FIGURE 1:**
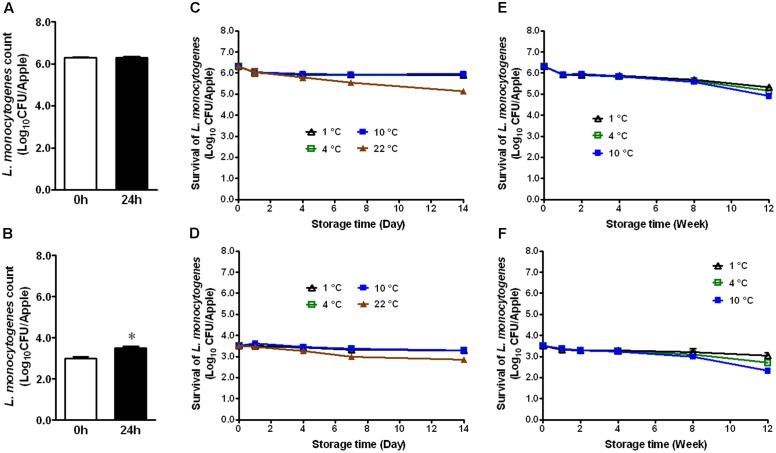
Survival of *Listeria monocytogenes* on fresh organic Granny Smith apples during short- and long-term storage. **(A,C,E)** Inoculation level of ∼6 Log_10_ CFU/apple; **(B,D,F)** Inoculation level of 3.5 Log_10_ CFU/apple. Mean ± SEM, *n* = 12.

Immediately after inoculation, organic GSA exhibited attachment of 6.29 ± 0.04 and 3.01 ± 0.06 Log_10_ CFU/apple of *L. monocytogenes*, respectively (**Figures 1A,B**). After 24 h attachment at RT, bacterial counts were 6.31 ± 0.06 and 3.51 ± 0.08 for their respective inoculation levels (**Figures 1A,B**). There was a significant increase of 0.5 Log_10_ CFU/apple for the low inoculation level (**Figure 1B**, *P* < 0.05). During 2 weeks of cold storage at 1, 4, and 10°C, bacterial populations did not significantly change (**Figures 1C,D**). On apples stored at 22°C, however, bacterial counts were reduced by 1.17 and 0.66 Log_10_ CFU/apple for the high and low inoculation levels, respectively (**Figures 1C,D**). *L. monocytogenes* established on fresh apples at either high or low level remained stable during cold storage. At both inoculation levels, only a 0.5–1.5 Log_10_ CFU/apple reduction on organic GSA during the 3-month cold storage was achieved (**Figures 1E,F**).

### *L. monocytogenes* Survival on Conventional GSA and Fuji Apples

**FIGURE 2:**
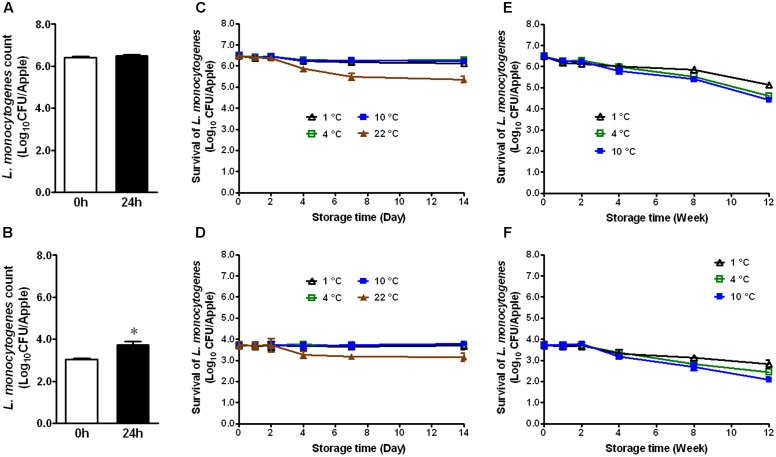
Survival of *L. monocytogenes* on fresh conventional Granny Smith apples during short- and long-term storage. **(A,C,E)** Inoculation level of ∼6 Log_10_ CFU/apple; **(B,D,F)** Inoculation level of 3.5 Log_10_ CFU/apple. Mean ± SEM, *n* = 12.

**FIGURE 3:**
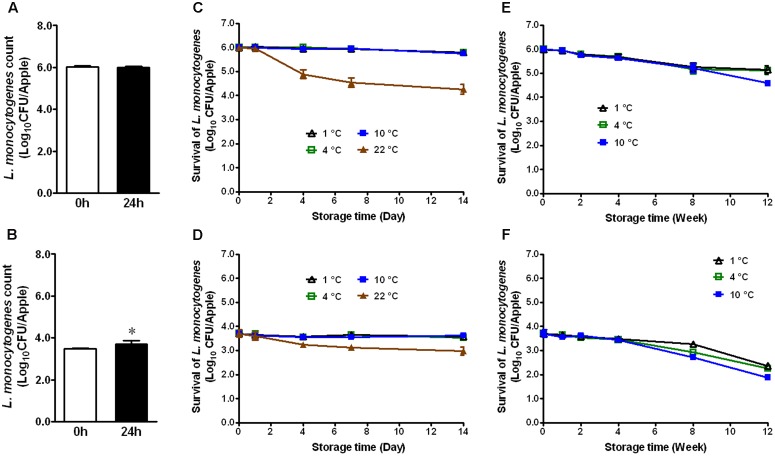
Survival of *L. monocytogenes* on fresh conventional Fuji apples during short- and long-term storage. **(A,C,E)** Inoculation level of ∼6 Log_10_ CFU/apple; **(B,D,F)** Inoculation level of 3.5 Log_10_ CFU/apple. Mean ± SEM, *n* = 12.

Apples from different agricultural practice or different varieties may behave differently in terms of bacterial adhesion and inactivation of the attached microorganisms to their surface. Therefore, we evaluated the fate of *L. monocytogenes* established on conventional GSA during storage. At the high inoculation level, *L. monocytogenes* maintained the same population size 24 h post-inoculation (**Figure 2A**). At the low inoculation level, the bacterial count was increased by ∼0.7 Log_10_ CFU/apple 24 h post-inoculation (**Figure 2B**, *P* < 0.05). After 2 weeks of cold storage, *L. monocytogenes* counts were slightly decreased for the high inoculation level, while no reduction was observed for the low inoculation level (**Figures 2C,D**). *L. monocytogenes* counts on apples stored at RT were reduced by 1.1 and 0.6 Log_10_ CFU/apple for high and low inoculation level, respectively (**Figures 2C,D**). After 3-month cold storage, a 0.9–2.0 Log_10_ CFU/apple reduction was achieved for both inoculation levels with 1°C showing the least reduction and 10°C the most (**Figures 2E,F**). Given the similar behavior of *L. monocytogenes* on organic and conventional GSA, the fate of *L. monocytogenes* was only accessed on conventional Fuji apples. Similar results were observed on inoculated conventional Fuji apples (**Figure 3**). After 2 weeks of cold storage, *L. monocytogenes* population on fresh Fuji apples was slightly reduced. There was about ∼1.7 and 0.7 Log_10_ CFU/apple reduction of *L. monocytogenes* on Fuji apples with high and low inoculation level, respectively, after 2-week of RT storage (**Figures 3C,D**), a slightly higher reduction than that on GSA. After 3-months, *L. monocytogenes* populations on Fuji apples were decreased by 0.8, 0.9, and 1.4 Log_10_ CFU/apple at 1, 4, and 10°C, respectively, for the high inoculation level (**Figure 3E**). For the low inoculation level, *L. monocytogenes* counts on Fuji apples declined by 1.4, 1.5, and 1.8 Log_10_ CFU/apple at 1, 4, and 10°C, respectively (**Figure 3F**).

### Evolution of Spoilage Microbiota in GSA and Fuji Apples

**FIGURE 4:**
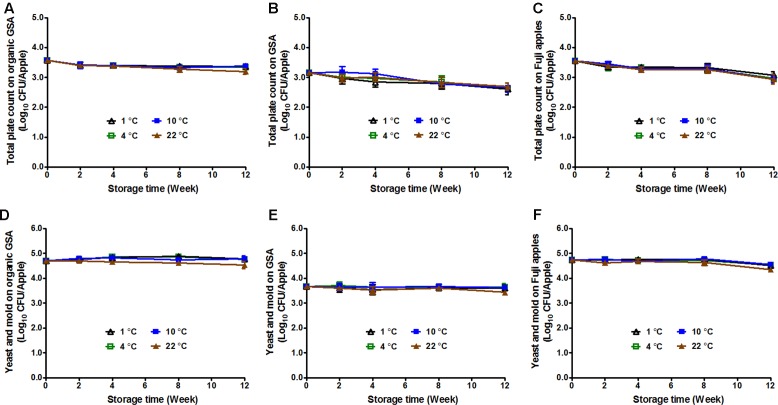
Fruit decay during 3-month storage at different temperatures. Total plate count **(A,B,C)** and yeasts/molds count **(D,E,F)** were evaluated. **(A,D)** Organic Granny Smith apples; **(B,E)** Conventional Granny Smith apples; **(C,F)** Conventional Fuji apples. Mean ± SEM, *n* = 12.

Bacteria, mold, and yeast can cause postharvest decay of apples (Janisiewicz and Korsten, 2002), reducing apple quality and causing a potential chemical (mycotoxin) hazard. Storage temperature has been reported to play a role in fruit decay (Mir et al., 2001). Therefore, TPC, in addition to Y/M count was performed on uninoculated apples of the selected varieties stored under different temperatures. The initial TPC was 3.57 ± 0.06, 3.16 ± 0.07, and 3.55 ± 0.06 Log_10_ CFU/apple for organic GSA, conventional GSA, and conventional Fuji apples, respectively (**Figures 4A–C**). After 3-month storage, TPC was significantly reduced by 0.2–0.4 Log_10_ CFU/apple for organic GSA, and 0.5–0.6 Log_10_ CFU/apple for conventional GSA and Fuji apples (**Figures 4A–C**). There was no difference in TPC on apples stored under different temperatures.

The initial Y/M count of organic GSA, conventional GSA, and conventional Fuji apples were 4.74 ± 0.05, 3.67 ± 0.10, and 4.73 ± 0.07 Log_10_ CFU/apple, respectively (**Figures 4D–F**). For both GSA (organic and conventional) and Fuji apples, Y/M count stayed the same throughout the cold storage with only a slight decline on apples stored at 22°C (**Figures 4D–F**).

## Discussion

### *L. monocytogenes* Attachment on Apples

At the inoculation level of ∼6 Log_10_ CFU/apple, *L. monocytogenes* population on fresh apples did not change during the 24 h establishment on the apple surface at RT, regardless of apple varieties or agricultural practice. Our results were corroborated with other previously published studies where the population of *L. monocytogenes* inoculated on whole strawberries at ∼7.5 Log_10_ CFU/berry was not significantly changed 24 h post-inoculation at 21°C (Flessa et al., 2005). Interestingly, at the low inoculation level, the population size of *L. monocytogenes* on apples slightly increased. Similarly, *L. monocytogenes* inoculated at ∼3 Log_10_ CFU/g on whole tomatoes were increased by ∼1.5 Log_10_ CFU/g after 2-day storage at 21°C (Beuchat and Brackett, 1991). While the mechanism for the observed difference between high and low inoculation levels is unknown, it might be due to the competition for the limited nutrients on apple surface at the high inoculation level.

### *L. monocytogenes* Population on Fresh Apples Decreased during Room Temperature Storage

A consumer survey study indicated that 42% of consumers have been reported to store apples at RT (Li-Cohen and Bruhn, 2002). Therefore, we evaluated behavior of *L. monocytogenes* on fresh apples during 2-week storage at RT. *L. monocytogenes* on apples of different cultivation practices (organic and conventional) or different varieties (GSA and Fuji apples) behaved similarly with 0.5–1.7 Log_10_ CFU/apple reduction regardless of the inoculation level after 2-week storage under RT. Consistent with our findings, *L. monocytogenes* inoculated onto a GSA surface at ∼5 Log_10_ CFU/apple was reduced by ∼1.5 Log_10_ CFU/apple after 15-day storage at 25°C (Salazar et al., 2016). The population of *L. monocytogenes* on whole strawberries at 7.1 Log_10_ CFU/berry was declined by 1.0 Log_10_ CFU/berry after 2-day storage under 24°C (Flessa et al., 2005). There was a 1.5 Log_10_ CFU/cm^2^ reduction of *L. monocytogenes* inoculated on whole cantaloupe surfaces at ∼3.5 Log_10_ CFU/cm^2^ during a 15-day storage at 20°C (Ukuku and Fett, 2002).

### *L. monocytogenes* on Fresh Apples during Cold Storage

Fresh apples are commercially stored at ∼1°C for extended periods of time (Mattheis et al., 1991). Apples are also held at 4°C in the packing house and consumer’s refrigerators at home (Duvenage and Korsten, 2016). During the transportation, apples may be exposed to temperature abuse of 10°C (Huang et al., 2015). Therefore, all three temperatures were tested in this study for extended storage of 3 months. Our results showed that *L. monocytogenes* on apples was stable during the first 2 weeks at all three temperatures regardless of inoculation level, apple variety, or growing practice. A similar phenomenon was observed when *L. monocytogenes* (5 Log_10_ CFU/g) was inoculated onto whole Golden Delicious apples; there was no *L. monocytogenes* reduction during a 9-day storage at 4°C (Rodgers et al., 2004). Similarly, the population size of *L. monocytogenes* inoculated on whole tomatoes at ∼3.5 Log_10_ CFU/g remained at the same level throughout the 21-day storage at 10°C (Beuchat and Brackett, 1991).

Survival of *L. monocytogenes* was similar among 1, 4, and 10°C, which was higher than that of RT storage. It might because apples stored at RT produced more volatile compounds including those with antimicrobial effects such as alcohols, aldehydes, ketones, and esters compared with apples at refrigerated storage (HamiltonKemp et al., 1996; Matich et al., 1996; Dixon and Hewett, 2000), which facilitated *L. monocytogenes* death under RT storage. On molecular level, cold storage might increase survival through altering fatty acid composition of the bacterial membrane (Mastronicolis et al., 2005), which protects *L. monocytogenes* against other environmental stresses during storage (Lou and Yousef, 1997). Cold temperatures may have also induced the expression of general stress sigma factor σ^B^, facilitating *L. monocytogenes* survival (Hecker and Volker, 1998; Becker et al., 2000). However, *L. monocytogenes* inoculated on whole strawberries was reduced by ∼3 Log_10_ CFU/berry after 7-day storage at 4°C (Flessa et al., 2005). These data indicate that a different food matrix and/or surface properties impacts *L. monocytogenes* survival at refrigerated temperature. The reduction of *L. monocytogenes* in strawberries might also be associated with the high moisture and low pH value of the fruit surface.

During 3 months of extended cold storage, reduction of *L. monocytogenes* on apples was temperature-dependent with relatively more reduction at 10°C. In support of our data, less *L. monocytogenes* were recovered from stainless steel surfaces stored at 10°C than those at 4°C (Redfern and Verran, 2017). This might due to the induction of a more fierce stress response and accumulation of more low molecular weight solutes at lower temperatures (NicAogain and O’Byrne, 2016). The phenomenon could also resulted from temperature-dependent alteration in apple fruit quality during long-term storage (Mir et al., 2001) with more antimicrobial volatile compounds production at a higher cold storage temperature (Dixon and Hewett, 2000). Inoculation level, apple variety, or agricultural practice had minor influences on *L. monocytogenes* survival on apple surfaces during cold storage.

### Storage Temperatures Had No Effect on Apple Fruit Decay

Resident bacteria, yeasts, and molds on apples contribute to fruit decay (Janisiewicz and Korsten, 2002). Thus, their fate was further studied over storage. TPC and Y/M counts stayed at the similar level throughout the 3-month storage under different temperatures for apples of the selected varieties and practices. In support of our finding, mesophilic aerobic microorganism, and Y/M counts were not changed on raw carrots during 18-day storage at 5°C (Beuchat and Brackett, 1990). However, inconsistent with our results, TPC and Y/M counts of raw carrots were increased by ∼0.5 and 1.5 Log_10_ CFU/g, respectively, after the 7-day storage at 15°C (Beuchat and Brackett, 1990). In another study, TPC as well as Y/M counts on whole tomatoes increased during the 20-day storage at 10°C and the 8-day storage at 21°C (Beuchat and Brackett, 1991). The difference in background flora behavior might due to different crop surfaces. The low-moisture condition on apple surface in this study might prevent the growth of resident bacteria, yeasts, and molds.

## Conclusion

Short-term RT storage resulted in 0.5–1.7 Log_10_ CFU/apple reduction of *L. monocytogenes* on fresh apples. Growers and packers in the apple industry often assume that *L. monocytogenes* on apples will die off during long-term cold storage. However, a limited reduction of *L. monocytogenes* on apple surfaces occurred during 12 weeks of refrigerated storage regardless of inoculation levels, apple varieties, or agricultural practices. This indicates that cold storage alone is not enough for eradicating *L. monocytogenes* from fresh apple surfaces. Additional intervention steps or hurdles for removal of *L. monocytogenes* are urgently needed during fruit storage or thereafter, such as during the packing of the fruit, in the apple industry.

## Author Contributions

LS is the main person who performed the experiment and wrote the manuscript. KE helped with the experiment and manuscript revision. H-CT helped with the experiment. IH provided the experimental apples and helped revise the manuscript. M-JZ guided the experimental design, experiment, as well as the manuscript revision.

## Conflict of Interest Statement

The authors declare that the research was conducted in the absence of any commercial or financial relationships that could be construed as a potential conflict of interest.
